# Pre-Operative Assessment of Micronutrients, Amino Acids, Phospholipids and Oxidative Stress in Bariatric Surgery Candidates

**DOI:** 10.3390/antiox11040774

**Published:** 2022-04-13

**Authors:** Thorsten Henning, Bastian Kochlik, Paula Kusch, Matthias Strauss, Viktorija Jurić, Marc Pignitter, Frank Marusch, Tilman Grune, Daniela Weber

**Affiliations:** 1 Department of Molecular Toxicology, German Institute of Human Nutrition Potsdam-Rehbruecke (DIfE), 14558 Nuthetal, Germany; thorsten.henning@dife.de (T.H.); paula.kusch@uni-potsdam.de (P.K.); scientific.director@dife.de (T.G.); 2 Food4Future (F4F), c/o Leibniz Institute of Vegetable and Ornamental Crops (IGZ), 14979 Grossbeeren, Germany; 3 NutriAct-Competence Cluster Nutrition Research Berlin-Potsdam, 14558 Nuthetal, Germany; bastian.kochlik@dife.de; 4 Department of Nutrition and Gerontology, German Institute of Human Nutrition Potsdam-Rehbruecke, (DIfE), 14558 Nuthetal, Germany; 5 Institute of Nutritional Science, University of Potsdam, 14469 Potsdam, Germany; 6 Department of Physiological Chemistry, Faculty of Chemistry, University of Vienna, 1090 Vienna, Austria; matthias_strauss@univie.ac.at (M.S.); viktoria.juric@univie.ac.at (V.J.); marc.pignitter@univie.ac.at (M.P.); 7 Vienna Doctoral School in Chemistry (DoSChem), University of Vienna, 1090 Vienna, Austria; 8 Department of General and Visceral Surgery, Klinikum Ernst von Bergmann, 14467 Potsdam, Germany; frank.marusch@klinikumevb.de; 9 Deutsches Zentrum für Diabetesforschung (DZD), 85764 Neuherberg, Germany

**Keywords:** obesity, micronutrients, oxidative stress, amino acids, phospholipids, vitamins, plasma

## Abstract

Obesity has been linked to lower concentrations of fat-soluble micronutrients and higher concentrations of oxidative stress markers as well as an altered metabolism of branched chain amino acids and phospholipids. In the context of morbid obesity, the aim of this study was to investigate whether and to which extent plasma status of micronutrients, amino acids, phospholipids and oxidative stress differs between morbidly obese (*n* = 23) and non-obese patients (*n* = 13). In addition to plasma, malondialdehyde, retinol, cholesterol and triglycerides were assessed in visceral and subcutaneous adipose tissue in both groups. Plasma γ-tocopherol was significantly lower (*p* < 0.011) in the obese group while other fat-soluble micronutrients showed no statistically significant differences between both groups. Branched-chain amino acids (all *p* < 0.008) and lysine (*p* < 0.006) were significantly higher in morbidly obese patients compared to the control group. Malondialdehyde concentrations in both visceral (*p* < 0.016) and subcutaneous (*p* < 0.002) adipose tissue were significantly higher in the morbidly obese group while plasma markers of oxidative stress showed no significant differences between both groups. Significantly lower plasma concentrations of phosphatidylcholine, phosphatidylethanolamine, lyso-phosphatidylethanolamine (all *p* < 0.05) and their corresponding ether-linked analogs were observed, which were all reduced in obese participants compared to the control group. Pre-operative assessment of micronutrients in patients undergoing bariatric surgery is recommended for early identification of patients who might be at higher risk to develop a severe micronutrient deficiency post-surgery. Assessment of plasma BCAAs and phospholipids in obese patients might help to differentiate between metabolic healthy patients and those with metabolic disorders.

## 1. Introduction

Prevalence of obesity (body mass index (BMI) ≥ 30 kg/m^2^) has risen worldwide over the last decades and is predicted to further increase, resulting in an estimated one in five adults to be obese by 2030 [1]. The causes of obesity are multifactorial, including psychological, genetic and environmental and socioeconomic factors [2,3,4,5]. Obesity is associated with increased risks for diseases such as type 2 diabetes, cardiovascular diseases, fractures and depression [6]. Deficiencies of micronutrients, especially iron, folate, vitamin B_12_ and vitamin D, are also associated with obesity [7,8,9]. Nonetheless, there is a subgroup of obese individuals who do not show an increased risk for obesity-related comorbidities, being described as metabolic healthy obese [10]. Metabolic profiling including screening of amino acids, acyl-carnitines and phospholipids has been used to differentiate between metabolic healthy obese (MHO) and metabolic unhealthy obese patients (MUHO), clearing the way for possible new therapeutic approaches to specifically target MUHO patients [11,12,13].

Established therapeutic approaches for weight reduction in obese patients range from dietary intervention and exercise, meal replacement and drug treatment to surgical intervention [14]. Bariatric surgery is reasonable for patients with a BMI of >40 kg/m^2^ or >35 kg/m^2^ with additional comorbidities who were unsuccessful with conventional therapies [14]. Malabsorption of micronutrients has been described following bariatric surgery [15,16]. Thus, morbidly obese patients undergoing surgery are at risk of exacerbating already existing micronutrient deficiencies [15]. Therefore, current clinical guidelines recommend assessing the nutritional status prior to bariatric surgery as well as supplementation of micronutrients post-surgery [14]. Furthermore, obesity is associated with increased oxidative stress due to increased formation of reactive oxygen species and decreased serum concentrations of antioxidant micronutrients [17]. A panel of redox biomarkers is associated with age, sex and obesity, including protein oxidation, lipid peroxidation and antioxidants [18]. Abdominal obesity in particular is a risk factor for obesity-associated secondary diseases. In this context, visceral adipose tissue (VAT) seems to be of central importance, as it is metabolically more active than subcutaneous adipose tissue (SAT) [19]. We hypothesize that concentrations of antioxidative lipid-soluble micronutrients are higher in VAT than in SAT and that plasma levels of fat-soluble micronutrients, oxidative stress, amino acids and phospholipids in morbidly obese patients differ from non-obese patients.

## 2. Materials and Methods

Patients with obesity-associated diseases who enrolled for bariatric surgery at the Ernst-von-Bergmann Clinic in Potsdam, Germany and had a body mass index of ≥35 kg/m^2^ were considered as subjects for the study group (morbidly obese patients, *n* = 23). The requirement for bariatric surgery was an unsuccessful weight loss therapy consisting of nutritional intervention, psychotherapy and sports therapy. The control group consisted of patients undergoing a medically necessary surgery and who had a BMI between 20 and 30 kg/m^2^ (*n* = 13). Patients with cancer were excluded from the study. EDTA plasma (4.9 mL) and serum (2.6 mL) were collected from participants in both groups within 24 h prior to the surgery. Plasma and serum were transferred and stored at the German Institute of Human Nutrition at −80 °C until analyses. Bioimpedance analysis was conducted one day before surgery in a lying down position using an Akern BIA 101 (SMT medical GmbH & Co. KG, Wuerzburg, Germany). Collection of VAT and SAT was carried out only if possible without additional surgical effort or risk for the patient, as part of the regular surgical procedure. VAT was obtained by surgical splitting of the peritoneum, while SAT was collected from the stitch canal of the trocar. Extracted tissue pieces of approximately 1 cm^3^ in size were then snap frozen in liquid nitrogen until further analysis. A lifestyle questionnaire including self-reported physical activity, educational status and smoking behavior was conducted in both groups a week before surgery. Additionally, participants in the obese group filled out a 4-day dietary record prior to surgical intervention. Ethical approval was granted from the Ethics committee of the University of Potsdam, Germany (No. 23/2015). Written informed consent was provided by all participants.

### 2.1. Biomarker Analyses

#### 2.1.1. Cholesterol

Plasma and adipose tissue cholesterol were analyzed by an enzymatic method, described by Deeg et al. [20], using a Cobas Mira autoanalyzer (Roche, Mannheim, Germany).

#### 2.1.2. Fat-Soluble Micronutrients

The carotenoids lutein/zeaxanthin, β-cryptoxanthin, lycopene and α-, β-carotene, as well as the vitamins α-, γ-tocopherol and retinol, were simultaneously analyzed in plasma by high-performance liquid chromatography (HPLC) with UV and fluorescence detection, as previously described [21]. In brief, plasma (40 µL) was extracted with ethanol/n–butanol (1:1, 200 µL) containing β-apo-carotenal-methyloxime as an internal standard. After centrifugation (21,000× *g*, 15 min at 4 °C), 20 µL of the clear supernatant was analyzed on a Shimadzu Prominence HPLC (LC-20A, Shimadzu, Duisburg, Germany) with chromatographic conditions, as previously described in detail [21]. Adipose tissue was prepared as follows: 150 mg frozen tissue were homogenized in 1.8 mL PBS with a ball mill. To saponify the lipids, 750 µL of the homogenate were stirred in the dark with 1 mL ethanol (containing the internal standard) and 500 µL KOH for 2.5 h at 40 °C. Then, 1 mL NaCl (15%, *w*/*v*) and 1 mL hexane were added, vigorously shaken and the upper phase transferred into a glass tube. This step was repeated with 1 mL hexane and upper organic phases combined. To clean the sample, 4 mL NaCl (7.5%, *w*/*v*) were slowly drizzled over the organic fraction and the supernatant again transferred into a clean glass tube. Samples were dried under nitrogen for about 25 min at room temperature. Samples were resuspended with 50 µL ethanol and 200 µL acetonitrile, centrifuged and supernatant was transferred into a HPLC vial for analysis, as described above.

#### 2.1.3. 25-Hydroxyvitamin D3

Frozen plasma was thawed on ice and an aliquot of 50 µL was mixed with 10 µL of an internal standard solution (1.36 µM d6-25OH-D3) and vortexed. To support the release of 25-hydroxyvitamin D3 from its protein-bond, 25 µL of 2M NaOH was added and samples were incubated for 20 min at room temperature. Protein precipitation was achieved by adding 300 µL of acetonitrile/methanol 9:1 (*v*/*v*). Samples were centrifugated for 10 min at 17,000× *g* at 4 °C. Solid Phase Extraction was performed using Oasis PRiME HLB 1 cc, 30 mg cartridges. First columns were equilibrated by 1 mL methanol followed by 1 mL MilliQ water. Samples were diluted with 750 µL MilliQ water and loaded onto the column. Subsequently, cartridges were washed with 1 mL of MilliQ water and dried for 30 s by applying negative pressure. Samples were eluted with 1 mL acetonitrile and evaporated to dryness for 1 h using a vacuum centrifuge. The dried samples were reconstituted in 50 µL of 1:1 methanol/MilliQ water (*v*/*v*) and transferred into amber vials with 100 µL glass inserts. Then, 5 µL were injected into the LC-system. Chromatography for 25-hydroxyvitamin D3 was performed using an ACQUITY UPLC BEH C18 column (2.1 mm × 50 mm, 1.7 µm) equipped with a VanGuard BEH C18 pre-column (2.1 mm × 5 mm, 1.7 µm). Mobile phase A consisted of MilliQ water containing 0.25% (*v*/*v*) formic acid while mobile phase B consisted of methanol containing 0.25% formic acid. Gradient elution was performed from 40% to 100% B in 3.5 min. Thereafter, strongly retained matrix compounds were washed off the column by keeping B at 100% for 0.5 min. Afterwards, the column was equilibrated with initial conditions (40% B) for 2 min. Flow rate was set at 0.25 mL/min. Samples were kept in the autosampler at 6 °C while column temperature was set at 50 °C. The mass spectrometer, a Xevo TQ-XS (Waters Corporation, Eschborn, Germany), was operated in ESI-positive mode. Desolvation temperature was set at 400 °C and desolvation gas flow was set at 600 L/h. The source capillary voltage was set at 3.0 kV, cone gas flow was set at 150 L/h and nebulizer gas pressure was set at 7.0 bar. Mass transitions with optimal collision energies for 25-hydroxyvitamin D3 measurements are shown in Supplemental Table S1.

#### 2.1.4. Malondialdehyde

Plasma malondialdehyde (MDA) was analyzed by reversed-phase (RP)-HPLC coupled with fluorescence detection after derivatization with thiobarbituric acid, as described by Wong et al. [22] with modifications [21]. Homogenized adipose tissue samples were processed like plasma samples by using 50 µL of homogenates instead of plasma (see section on Fat-soluble micronutrients).

#### 2.1.5. Protein Carbonyls and 3-Nitrotyrosine

Plasma concentrations of protein carbonyls and protein-bound 3-nitrotyrosine were measured by non-commercial in-house ELISA methods, as previously described [21].

#### 2.1.6. Amino Acids

Ten µL of plasma were mixed with 40 µL of internal standard working solution containing eleven deuterated amino acids (62.5 µM in 90% acetonitrile, see Supplemental Table S2) and vortexed for 30 s. To support protein precipitation, samples were stored at −20 °C for 10 min in a precooled rack and then centrifuged at 30.000× *g* and 4 °C for 10 min. Next, 40 µL of supernatant were transferred into a vial with a 100 µL glass insert and 2 µL were injected into the LC system. Separation of amino acids was performed using an ACQUITY UPLC BEH Amide column (2.1 mm × 100 mm, 1.7 µm) equipped with a VanGuard BEH Amid pre-column (2.1 mm × 5 mm, 1.7 µm). Mobile phase A consisted of acetonitrile/MilliQ water (9:1, *v*/*v*) containing 5 mM ammonium formate and 0.3% formic acid (*v*/*v*). Mobile phase B consisted of MilliQ water containing 25 mM ammonium formate (pH 6). Gradient elution started at 98% A and was held for 4 min. Thereafter, the proportion of solvent B was increased to 25% within 0.5 min. To start, 25% B was maintained between 4.5 and 6.5 min and then solvent B was increased to 40% within 0.1 min. The 40% B solvent was kept constant between 6.6 and 8.0 min. Finally, solvent B was decreased to 2% within 0.1 min and the column was re-equilibrated with initial conditions for another 1.9 min, resulting in a total runtime of 10 min. The flow rate was constantly set at 0.4 mL/min. Samples were kept in the autosampler at 6 °C and column temperature was set at 35 °C. The mass spectrometer, a Xevo TQ-MS (Waters Corporation, Eschborn, Germany), was operated in ESI-positive mode. Desolvation temperature was set at 600 °C and desolvation gas flow was set at 600 L/h. The source capillary voltage was set at 0.5 kV, cone gas flow was set at 150 L/h and nebulizer gas pressure was set at 7.0 bar. Mass transitions for each amino acid are shown in Supplemental Table S2.

#### 2.1.7. Phospholipids

For the analysis of plasma phospholipids, samples were extracted according to the protocol provided for the SPLASH^®^ LIPIDOMIX^®^ standard from Avanti Polar Lipids (Alabaster, AL, USA). After thawing on ice, 10 μL plasma was added to 990 μL water. The samples were vortexed, then incubated on ice for 10 min. After the addition of 2 mL methanol and 0.9 mL dichloromethane, 10 μL SPLASH™ internal standard was transferred to each tube. The samples were vortexed again and the mixture was incubated for 30 min at room temperature. A total of 1 mL water and 0.9 mL dichloromethane was added to each tube and the tubes were inverted 10 times. The samples were centrifuged at 1200 rpm for 10 min. Each lower layer was collected and transferred into a new tube. Dichloromethane (2 mL) was added to the remaining upper phases in the extraction tubes, which were then shaken and centrifuged. The lower layers were collected and added to first extracts. The solvent was evaporated under a gentle stream of nitrogen. Prior to LC-MS analysis, the samples were resuspended in 100 µL isopropanol. The ThermoFischer Scientific Vanquish LTQ Orbitrap Velos was connected with a Phenomenex Kinetex^®^ 2.6 µm C18 100 Å (150 × 2.1 mm) column. Solvent A was acetonitrile:H_2_O (60:40, *v*/*v*) and solvent B consisted of isopropanol:acetonitrile:H_2_O (90:8:2, *v*/*v*/*v*); 10 mM ammonium formate and 0.1% (v) formic acid was added to both solvents. A gradient according to Ulmer et al. [23] was used with slight modifications: 32% B at 0 min, 40% B at 1 min, hold at 40% until 1.5 min, 45% B at 5 min, 50% B at 6 min, 60% B at 10 min, 70% B at 13 min and 80% B at 18 min, followed by 5 min equilibration at 32% B. The flow rate was 0.3 mL/min. The ESI was operating with the following parameters: source heater temperature set to 400 °C, capillary temperature set to380 °C, sheath gas flow and auxiliary gas flow and sweep gas flow at 50, 20 and 2 arbitrary units, respectively. A voltage of 3.0 kV was applied in both positive and negative ion mode. Spectra were recorded in the mass range of m/z 100–1000. Quality control was assured by randomization of the sequence and utilization of a quality control sample, which was pooled from all samples. The pooled sample was injected five times before the actual sequence of samples and then after every fifth sample. MS-DIAL software (v. 4.80, RIKEN) was used to process the MS data [24]. Default parameters were used, except minimum peak height, which was set to 100,000. The peaks were annotated by accurate mass and MS/MS matching with the public LipidBlast library. The results were confirmed by further matching with the human metabolome database [25]. The data were normalized by utilizing the class-specific internal standards. The detected phospholipids were summed up for each lipid class and semi-quantified by comparing their area to the area of the internal standard of the respective lipid class.

### 2.2. Statistical Analysis

Demographic characteristics are described using means and SD for continuous variables (age, weight, height, body mass index (BMI (kg/m^2^); fat mass, muscle mass, screen time including time spent in front of computer and television, physical activity, cigarettes; see Table 1) and frequencies (% (*n*)) for categorical variables (sex, working status, smoking status). Differences in characteristics and biomarkers between study groups were compared by independent sample *t*-test (continuous variables) and Pearson’s chi-squared test (prevalence); differences between SAT and VAT within study groups were assessed by paired samples *t*-test. Biomarker data were transformed to achieve normal distribution using square root (SR), inverse (1/X) or logarithmic (LN) transformation as appropriate and are described by geometric means with 95% confidence intervals (CI). Statistically significant differences were considered to be present at *p* < 0.05. All statistical analyses were carried out using SPSS software (SPSS Inc., Chicago, IL, USA; Version 25). Statistical analysis for phospholipids was conducted in SigmaPlot (Inpixon GmbH, Düsseldorf, Germany; Version 14.5). All data were examined for normal distribution by the Shapiro–Wilk test and for equal variance by the Brown–Forsythe test. Student’s *t*-test was performed to identify differences in phospholipid concentrations between the obese and the control group. In case the Brown–Forsythe test failed, Welch’s *t*-test was performed instead. In case the Shapiro–Wilk test failed, Mann–Whitney U test was applied. Nalimov tests were performed to check for outliers.

## 3. Results

Characteristics of the study’s participants are shown in Table 1. Participants were grouped according to their BMI and whether bariatric (obese) or other surgery (control) was performed. The obese group displayed an average BMI of 46.3 ± 7.0 kg/m^2^, where the control group showed a BMI of 26.2 ± 2.4 kg/m^2^. The obese group was significantly younger and had a significantly higher proportion of men, as well as a higher muscle mass compared to the control group. Lifestyle behaviors regarding both weekly times spend with exercise and screen time were similar in both groups. Working and smoking status were also similar in both groups.

### 3.1. Biomarker Concentrations in Plasma Samples

Obese participants had significantly lower plasma concentrations of γ-tocopherol than control patients. Plasma values for α-tocopherol and α-carotene, β-carotene, total lutein/zeaxanthin, β-cryptoxanthin and lycopene as well as retinol and 25-hydroxyvitamin D3 were similar in both groups (see Table 2).

Regarding plasma concentrations of oxidative stress markers 3-nitrotyrosine, MDA and protein carbonyls, no differences between both groups were observed (see Table 3).

Plasma concentrations of branched-chain amino acids leucine, isoleucine and valine were significantly higher in the obese group compared to the control group. While plasma lysine was also higher in the obese group, lower levels of 1-methyhistidine were observed compared to the control group (see Table 4).

### 3.2. Biomarker Concentrations in Adipose Tissue

Concentrations of MDA were significantly higher in the obese group both in VAT and SAT compared to the control group (see Table 5). Retinol was significantly higher in the SAT of obese compared to SAT of control patients. Furthermore, retinol was significantly higher in SAT than in VAT within the obese group. None of the other biomarkers differed within groups between SAT and VAT.

We found significant correlations between total body fat mass and some biomarkers (Supplemental Table S3). For instance, there was a strong positive correlation between fat mass and MDA in VAT and in SAT (see Supplemental Figure S1), but not with plasma MDA. In contrast, fat mass correlated inversely with plasma retinol while SAT-retinol was positively associated and VAT-retinol did not correlate with fat mass at all. Plasma β-carotene and 1-methylhistidine were also inversely associated with fat mass while lysine was positively associated.

The only biomarkers that we were able to analyze in tissue were retinol, MDA, cholesterol and triglycerides; the other biomarkers were below the limit of detection. We found that MDA of both SAT and VAT strongly correlated with each other (r = 0.723, *p* = 0.002).

Concerning the analysis of lipid-metabolism-related biomarkers, phosphatidylcholines (PC), lyso-phosphatidylcholines (LPC), phosphatidylethanolamines (PE), lyso-phosphatidylethanolamines (LPE) and phosphatidylinositols (PI), and additionally ether-linked PC O, PE O, LPC O and LPE O, were detected and semi-quantified. The concentrations of PC, PE, LPE and ether-linked PC O, PE O and LPE O were significantly lower in obese participants (Table 6).

## 4. Discussion

Micronutrient deficiency in morbidly obese persons may seem paradoxical due to the high food intake, but consistent observations have been made on this phenomenon by different research groups. A link between obesity and reduced levels of serum or plasma carotenoids, retinol and tocopherols has been reported in several observational studies [17,26,27,28]. In our study, vitamin A deficiency (cut-off value in plasma <0.75 µM) was found in 21.7% of the obese group and in 7.7% of the control group (see Table 7). In contrast, Ernst et al. found no vitamin A deficiency in obese patients using the same cut-off value [29]. The prevalence of vitamin A deficiency measured by plasma levels in bariatric surgery candidates ranged between 0 and 2.7%, using cut-off values of either <1.05 [30,31] or <1.2 µM [32]. Lefebvre et al. reported a prevalence of vitamin A deficiency of 16.9%; however, their cut-off value < 1.63 µM was higher [33]. The prevalence for vitamin E deficiency (cut-off < 11.6 µM) in the morbidly obese group was 8.7%, and thus, even lower than in the control group with 15.4%. While the prevalence of vitamin E deficiency in obese patients was reported as ranging from 0 to 2.2% [29,31] using similar cut-off values (<12 µM), up to 20% of obese men and women showed vitamin E deficiency when adjusted for total lipids (<3.6 µmol/mmol) [32].

A higher risk for vitamin D deficiency (<50 nM) was also found to be associated with obesity [34]. In addition, vitamin D levels were found to further decrease in follow-up visits when left untreated [16]. Adequate pre-surgery vitamin D is associated with improved insulin resistance post-surgery independent of metabolic phenotype [35]. Perhaps measuring vitamin D levels some weeks prior to surgery and supplementation could minimize the risk for developing chronic diseases.

Furthermore, a link between low vitamin D status and low levels of insulin regulating adipokine resistin has been proposed [36]. We found 52.2% of obese participants had a moderate vitamin D deficiency (<50 nM) and 13.0% showed a severe deficiency (<25 nM). The prevalence of moderate vitamin D deficiency in bariatric surgery candidates was reported from 47.0 to 67.9% (<50 nM) [29,33,37,38,39]. Data on severe vitamin D deficiency among obese patients depend on the selected cut-off values and range from 10.9–25.4% [29,32,33] (<25 nM) to 19–26.1% (<37.5 nM) [30,31]. It has been shown that by adequate supplementation of micronutrients, the risk of deficiency, caused by malabsorption after surgery, can be prevented [40]. However, the adherence to recommended supplementation is low (approximately 30% six months after surgery) [41]. It is promising that information from dieticians at follow-up visits was able to increase adherence to supplementation [42,43]. Therefore, supplementation should be accompanied by nutritional counselling in order to increase adherence. In our current study, in addition to lifestyle factors, it is also possible that polymedication contributed to micronutrient status as we previously showed in healthy participants [44]. Unfortunately, we did not assess medication intake in this study. Another limiting factor in our study is that information about possible vitamin supplementation as well as hours spent in the sun was not available.

In a recent meta-analysis, Iqbal et al. [45] identified an inverse relationship between serum carotenoids with obesity and metabolic diseases. We were expecting lower carotenoids in the obese group but, based on the relatively small sample size, there were no differences in plasma carotenoids between our groups. We found that total carotenoid concentration in VAT correlated inversely with BMI (r = −0.555, *p* = 0.032) and fat mass (r = −0.586, *p* = 0.022), but SAT total carotenoids were not associated with BMI or with fat mass (data not shown). These results correspond with those of Harari et al., who also found that [46] multiple serum and adipose tissue carotenoids were associated with favorable metabolic traits, including insulin sensitivity in liver and adipose tissue.

When comparing carotenoid concentrations measured in this study with another study from Germany [47], we found that our participants had quite similar concentrations of α- and β-carotene as participants in the EPIC Potsdam and EPIC Heidelberg cohort, whereas β-cryptoxanthin and lutein/zeaxanthin were lower and lycopene was higher in our study. However, these carotenoids are dependent on the season and, in the small sample, we cannot control for season, since participants were recruited throughout the year. Lower serum levels of retinol and α-tocopherol in obese patients have been discussed as being related to elevated markers of oxidative stress [17]. We found significantly lower plasma γ-tocopherol in the obese group, however, after adjusting for cholesterol, there were no differences (data not shown). Furthermore, there were no differences in the commonly used redox biomarkers in plasma, which is also contrary to the literature.

We did not observe any significant correlation between plasma biomarkers of oxidative stress and fat-soluble micronutrients (results not shown).

However, tissue concentrations of MDA were significantly higher in both SAT (four times higher) and VAT (three times higher) in the obese compared to the control group, but there was no difference between tissue types within study groups. This is in accordance with another study, where in lean, obese and obese patients with diabetes mellitus, no significant differences in VAT–MDA concentrations were observed [48]. Higher tissue concentrations of MDA may be a result of inflammatory and metabolic processes that are commonly observed in obesity [49]. We had expected that VAT, as an endocrine organ compared to SAT, would show higher concentrations of MDA, but the lack of statistical significance might be due to the small sample size.

There were significant differences in total BCAA concentrations between the obese group (489.5 ± 111.6 µM) and the control group (401.7 ± 80.5 µM) (*p* < 0.008) prior to surgery. Associations between elevated plasma BCCA levels and obesity have been previously described [50,51,52,53]. High plasma BCAA levels are linked to poor metabolic health [51] and are suggested to serve as a predictor for insulin resistance [52]. Therefore, measuring pre-surgery BCAA concentrations may support post-surgery therapeutic approaches. Pharmacological targeting of BCAA catabolism in obese mice was shown to ameliorate insulin resistance and hyperglycemia [54]. The cause for elevated plasma levels of BCAAs may result from impaired activity of BCAA metabolizing enzymes, observed in obesity [55,56]. Differences in plasma 1-methylhistidine (biomarker for meat intake) between our groups might be explained by meat consumption [57] the days before the surgery. Unfortunately, dietary data from the control group were not available.

Significant differences were observed in the plasma concentrations of PC, PE, LPE and the corresponding ether-linked analogs, which were all lower in obese participants compared to the control group. Obesity is associated with lower levels of plasma phospholipids including unsaturated fatty acids, such as FA 18:2 (linoleic acid), in phospholipids, as well as ether-linked plasma phospholipids, which is confirmed in the present study [13,58].

Limitations of our study are the small sample size and the fact that adipose tissue samples were not available from all participants. The small sample size did not allow subgroup analysis. Insulin and glucose measurements were not performed; thus, the HOMA-IR is missing and no discrimination between MHO and MUHO was made. As discussed above, we could not control for season, which is known to have an effect, especially on carotenoids and vitamin D. In addition, the consumption of dietary supplements such as multivitamins possibly also had an effect on our results. Data from the NHANES study show that 51% of participants consumed at least one dietary supplement (in the past 30 days) and supplement use was inversely associated with BMI [59], resulting in possibly higher supplement use in the control group. We cannot exclude selection bias. Participants in the control group were selected based only on the facts that a medically necessary surgery was being performed where adipose tissue could be removed, underlying disease was non-malignant and their BMI was between 20 and 30 kg/m^2^. The underlying disease will likely have had an effect on biomarker concentrations. Hospital patients are assumed to have different characteristics than the general population but, in this study, it was feasible due to logistical reasons. In addition, due to the mode of recruitment, the control group was not able to provide dietary records.

The strengths of this study include various up-to-date analytical methods and the broad range of biomarkers. In addition, we had the opportunity to measure adipose tissue samples (especially both types of adipose tissue with VAT and SAT) with corresponding plasma samples in obese and in control participants. We believe, to the best of our knowledge, that this broad panel of biomarkers, i.e., the combination of BCAA, micronutrients, redox and lipid metabolism biomarkers, has not been assessed in these kinds of patients before. Future studies should include at least one follow-up with blood sampling to analyze changes in biomarker/micronutrient status as well as changes in metabolic state. In addition, better metabolic characterization and a larger sample size is strongly recommended.

## 5. Conclusions

In this study, there were significant differences in plasma amino acids between the two study groups, with higher concentrations in the obese group representing a metabolic unhealthy phenotype. In contrast, there were little differences in fat-soluble micronutrient concentrations. Participants in the obese group had significantly lower γ-tocopherol plasma concentrations and higher retinol SAT concentrations. Concerning oxidative stress, we found higher MDA concentrations in the obese study group in both tissue types, which might be a sign of metabolically active tissue. Therefore, the hypothesis that there is an increased storage of lipid-soluble micronutrients in adipose tissue of obese subjects cannot be confirmed. This could indicate that these antioxidant lipid-soluble micronutrients might have been consumed in compensatory mechanisms due to oxidative stress and/or inflammation.

Pre-operative assessment of micronutrients is reasonable for an early identification of patients who might be at risk of developing a severe micronutrient deficiency after undergoing bariatric surgery. Clinical guidelines recommend preoperative micronutrient screening of bariatric surgery patients and lifelong supplementation of micronutrients post-surgery including iron, vitamin B_12_, folic acid, vitamin D, vitamin A and calcium, dependent on the type of operational procedure (e.g., Roux-en-Y gastric bypass, Laparoscopic bypass) performed [14]. Patients’ post-surgery micronutrient status should be assessed in follow-up visits on an annual basis to monitor adherence to any prescribed supplementation program. Additionally, higher fasting plasma values of total BCAAs as well as lower plasma phospholipid concentrations might serve as a potential marker for impaired metabolism and future metabolic diseases in obese patients [50].

## Figures and Tables

**Table 1:** Participant characteristics.

	Control (*n* = 13)	Obese (*n* = 23)	*p*-Value
Age (years)	61.00 ± 13.55	46.12 ± 10.26	0.001
Sex (male)	58.3 (7)	17.4 (4)	0.013
Weight (kg)	78.73 ± 8.57	129.51 ± 25.93	<0.001
Height (m)	1.73 ± 0.06	1.67 ± 0.10	0.025
BMI (kg/m^2^)	26.23 ± 2.36	46.34 ± 7.04	<0.001
Fat mass (kg)	21.04 ± 8.48	61.43 ± 18.59	<0.001
Muscle mass (kg)	35.09 ± 9.55	44.01 ± 10.82	0.037
Screen time (h/week)	25.20 ± 15.02	23.57 ± 16.07	0.841
Physical Activity (h/week)	3.09 ± 3.78	3.33 ± 2.50	0.540
Cigarettes (*n*/day)	1.82 ± 4.05	5.13 ± 8.76	0.443
Working, currently	45.5 (5)	57.1 (8)	0.561
Smoking, currently	18.2 (2)	35.7 (5)	0.332

Values are given as mean ± SD or percentage (*n*). Independent samples *t*-test for continuous variables, chi square test for categorical variables. *p* < 0.05.

**Table 2:** Plasma lipid-soluble micronutrient concentrations by study group.

	Control (*n* = 13)	Obese (*n* = 23)	*p*-Value
Lutein/Zeaxanthin (µM)	0.075 (0.039; 0.145)	0.050 (0.027; 0.093)	0.377
β-Cryptoxanthin (µM)	0.037 (0.020; 0.067)	0.040 (0.024; 0.068)	0.818
Lycopene (µM)	1.068 ± 0.582	0.927 ± 0.429	0.412
α-Carotene (µM)	0.118 (0.070; 0.179)	0.133 (0.092; 0.183)	0.675
β-Carotene (µM)	0.319 (0.189; 0.483)	0.247 (0.167; 0.342)	0.355
Retinol (µM)	1.444 (1.135; 1.838)	1.137 (0.878; 1.472)	0.209
α-Tocopherol (µM)	23.94 ± 8.02	19.49 ± 4.97	0.087
γ-Tocopherol (µM)	1.318 ± 0.577	0.874 ± 0.412	0.011
25-Hydroxyvitamin D3 (nM)	62.50 ± 32.09	53.76 ± 30.65	0.424

Values are given as mean ± SD or geometric mean with 95% CI. Independent samples *t*-test. *p* < 0.05.

**Table 3:** Plasma biomarkers of oxidative stress by study group.

	Control (*n* = 13)	Obese (*n* = 23)	*p*-Value
3-Nitrotyrosine (pmol/mg)	2.35 ± 1.09	2.51 ± 0.95	0.640
Protein carbonyls (nmol/mg)	0.251 ± 0.093	0.255 ± 0.118	0.929
Malondialdehyde (µM)	0.919 (0.705; 1.198)	0.784 (0.641; 0.863)	0.120

Values are given as mean ± SD or geometric mean with 95% CI. Independent samples *t*-test. *p* < 0.05.

**Table 4:** Plasma amino acid concentrations by study group.

	Control (*n* = 13)	Obese (*n* = 23)	*p*-Value
Leucine (µM)	117.5 ± 26.5	144.6 ± 37.1	0.027
Isoleucine (µM)	55.9 ± 11.8	68.8 ± 19.3	0.018
Valine (µM)	228.3 ± 42.3	276.6 ± 55.2	0.006
Total BCAA (µM)	401.7 ± 80.5	489.5 ± 111.6	0.008
3-Methylhistidine (µM)	6.0 ± 1.5	6.1 ± 2.7	0.884
1-Methylhistidine (µM)	4.78 (2.28; 10.03)	1.37 (0.79; 2.37)	0.008
Proline (µM)	174.8 ± 62.0	159.4 ± 48.1	0.411
Phenylalanine (µM)	59.3 ± 25.7	56.2 ± 10.3	0.690
Lysine (µM)	153.0 ± 28.0	186.1 ± 35.0	0.006
Histidine (µM)	64.5 ± 14.3	65.5 ± 8.4	0.833
Arginine (µM)	115.5 ± 42.6	90.0 ± 37.7	0.071
Threonine (µM)	85.7 ± 16.7	95.8 ± 26.0	0.219
Tryptophan (µM)	28.1 ± 7.0	27.4 ± 6.2	0.772
Tyrosine (µM)	60.5 ± 13.7	66.6 ± 15.9	0.256

Values are given as mean ± SD or geometric mean with 95% CI. Independent samples *t*-test. *p* < 0.05.

**Table 5:** Adipose tissue concentrations of biomarkers.

	Control (*n* = 7)	Obese (*n* = 8)	*p*-Value
***Visceral Adipose Tissue***			
MDA (µmol/kg)	2.77 (1.64; 4.67)	9.82 (4.02; 24.0)	0.016
Cholesterol (mmol/kg)	0.553 ± 0.330	0.875 ± 0.435	0.134
Triglycerides (mmol/kg)	3.74 ± 0.54	3.97 ± 0.57 ^1^	0.476
Retinol (µmol/kg)	5.83 ± 3.83	5.87 ± 3.06 ^#^	0.981
***Subcutaneous Adipose Tissue***			
MDA (µmol/kg)	4.03 (2.01; 8.07)	16.8 (12.8; 21.9)	0.002
Cholesterol (mmol/kg)	0.673 ± 0.567	0.895 ± 0.405	0.390
Triglycerides (mmol/kg)	0.346 ± 0.97	3.64 ± 0.40	0.460
Retinol (µmol/kg)	4.87 ± 3.86	10.64 ± 2.49	0.007

^1^ (*n* = 6), independent samples *t*-test (between groups), ^#^ *p* < 0.01, paired sample *t*-test (within groups comparison).

**Table 6:** Plasma cholesterol and phospholipids.

	Obese (*n* = 23)	Control (*n* = 13)	*p*-Value
Cholesterol (mM)	4.51 (1.07)	5.23 (1.06)	0.059
LPC (µM)	190.7 (46.00)	204.1 (66.99)	0.491
PC (mM)	2.67 ± 0.55	3.21 ± 0.57	0.007
PE (µM)	35.78 ± 10.42	45.05 ± 11.20	0.019
PI (µM)	0.94 ± 0.51	1.10 ± 0.40	0.344
LPE (µM)	11.66 ± 3.03	16.62 ± 7.51	0.038
LPC O (µM)	2.96 ± 1.10	4.13 ± 2.07	0.075
PC O (µM)	121.9 ± 27.66	148.1 ± 24.05	0.009
PE O (µM)	15.69 ± 8.10	25.69 ± 10.59	0.006
LPE O (µM)	0.44 ± 0.23	0.84 ± 0.32	<0.001

Values are given as mean ± SD. *p*-values that indicate significant difference between the groups are marked bold (Student’s *t*-test or Welch’s *t*-test in case of inequal variances; Mann-Whitney U test for PE O due to failed Shapiro-Wilk test).

**Table 7:** Prevalence of vitamin deficiency by study group.

	Control (*n* = 13)	Obese (*n* = 23)	*p*-Value
Vitamin A (Retinol) < 0.75 µM	7.7 (1)	21.7 (5)	0.382
Vitamin E (α-Tocopherol) < 11.6 µM	15.4 (2)	8.7 (2)	0.609
Vitamin D (25-OH D3) < 50 nM	38.5 (5)	52.2 (12)	0.502
Vitamin D (25-OH D3) < 25 nM	0 (0)	13.0 (3)	n.d.

% (*n*), chi-squared test.

## Data Availability

Data is contained within the article and Supplementary Material.

## Supplementary Materials

The following are available online at https://www.mdpi.com/article/10.3390/antiox11040774/s1, Table S1: Vitamin D analysis by UPLC-MS/MS; Table S2: Amino acid analysis by UPLC-MS/MS. Table S3: Correlations between fat mass and biomarkers, Figure S1: Correlation between MDA tissue concentrations and fat mass.
