# Supplementary material for: Pre-Operative Assessment of Micronutrients, Amino Acids, Phospholipids and Oxidative Stress in Bariatric Surgery Candidates
Source: Antioxidants (Basel). 2022 Apr 13;11(4):774. doi: 10.3390/antiox11040774 (PMC9031169; doi:10.3390/antiox11040774)

Supplemental Table S1: Vitamin D analysis by UPLC-MS/MS

MRM transitions and parameters for vitamin D analysis

Analyte	Retention time [min]	MRM Transition	Cone Voltage (V)	Collision Energy (eV)
25-OH-D3	4.98	401.3 > 365.3*	8	10
		401.3 > 159.1	8	26
		401.3 > 389.3	8	8
d6-25-OH-D3	4.98	407.3 > 371.3*	10	10
		407.3 > 389.3	10	8

* Quantifier

Accuracy:

Accuracy was assessed by analyzing NIST Standard Reference Material 972a and comparing the calculated concentrations with the certified values. Each reference value was measured as triplicate (Level 1-4).

Concentration Level	Certified Concentration [nM]	SD [nM]	Measured concentration [nM]	SD [nM]	Deviation [%]
1	76.3*	2.9	78.4	1.7	3.8
2	48.3*	1.2	52.2	1.0	2.9
3	52.4*	1.5	54.2	1.0	2.5
4	138.2*	7.7	144.9	2.8	5.6

*Values represent combined values of 25-OH-D3 and epi-25-OH-D3

Precision: For determination of intra-day and inter-day as well as inter-week precision plasma samples from three healthy individuals showing significantly different 25-OH-D3 serum levels were measured over a 3-week period.

Precision data for the LC-MS/MS analysis of 25-OH vitamin D_3						
	Intra-day (n =3)		Inter-day (n = 9)		Inter-week (n = 27)	
Concentration Level	Mean (SD), nmol/L	CV %	Mean (SD), nmol/L	CV %	Mean (SD), nmol/L	CV %
Low	13.4 (0.2)	1.5	12.9 (0.5)	3.7	13.0 (0.5)	4.1
Mid	35.8 (1.3)	3.7	36.5 (1.3)	3.5	37.8 (2.0)	5.4
High	63.7 (0.8)	1.3	62.4 (1.7)	2.8	64.0 (2.3)	3.7

Supplemental Table S2: Amino acid analysis by UPLC-MS/MS

MRM transitions and parameters for AA analysis

Analyte	Retention time [min]	MRM Transition	Internal Standard (IS)	Cone Voltage (V)	Collision Energy (eV)
Leucine	3.36	132.1 > 86.1	Leucine-$^{13}C_6$, N^{15}	20	10
Leucine-$^{13}C_6$, N^{15}	3.36	139.1 > 92.1		22	8
Phenylalanine	3.38	166.1 > 120.1	Phenyl-$^{13}C_6$-alanine	12	12
Phenyl-$^{13}C_6$-alanine	3.38	172.1 > 126.1		12	14
Tryptophan	3.53	205.1 > 188.1	Phenyl-$^{13}C_6$-alanine	10	10
Isoleucine	3.68	132.1 > 86.1	Isoleucine-$^{13}C_6$, N^{15}	20	10
Isoleucine-$^{13}C_6$, N^{15}	3.68	139.1 > 92.1		22	8
Methionine	4.54	150.0 > 56.0	Methionine-$^{13}C_5{}^{15}N$	20	14
Methionine-$^{13}C_5{}^{15}N$	4.54	156.0 > 60.0		24	14
Valine	4.91	118.1 > 72.0	Valine-$^{13}C_5$	14	10
Valine-$^{13}C_5$	4.91	123.1 > 76.0		16	8
Proline	5.24	116.0 > 70.1	Proline-$^{13}C_5$	20	12
Proline-$^{13}C_5$	5.24	121.1 > 74.0		22	12
Threonine	5.73	120.1 > 74.0	Threonine-$^{13}C_4$	14	10
Threonine-$^{13}C_4$	5.73	124.1 > 77.0		16	8
3-Methylhistidine	6.23	170.1 > 124.0	3-Methylhistidine-d3	10	14
3-Methylhistidine-d3	6.23	173.1 > 127.0		10	14
1-Methylhistidine	6.52	170.1 > 96.1	3-Methylhistidine-d3	10	18
Histidine	6.53	156.1 > 110.1	Histidine-$^{13}C_6$	10	14
Histidine-$^{13}C_6$	6.53	162.1 > 115.1		10	14
Arginine	6.53	175.1 > 70.1	Arginine-$^{13}C_6$	32	18
Arginine-$^{13}C_6$	6.58	181.2 > 74.0		30	20
Lysine	6.67	147.0 > 84.0	Lysine-$^{13}C_6$	10	16
Lysine-$^{13}C_6$	6.67	153.1 > 89.0		10	16

Accuracy: Accuracy was assessed by measuring NIST SRM 1950 and comparing the calculated concentrations with the certified concentrations. Measured concentrations and standard deviations are expressed as a mean resulting from 24 measurements.

Accuracy for AA measurements					
Analyte	Certified concentration [µM]	SD [µM]	Measured concentration [µM] (mean n=24)	SD [µM]	Deviation [%]
Leucine	100.4	6.4	95.5	9.8	- 4.9
Phenylalanine	51.0	7.0	49.4	3.2	-3.2
Isoleucine	55.5	3.4	49.6	5.1	-10.7
Methionine	22,3	1.8	20.1	1.6	-9.9
Valine	182.2	10.4	181.8	15.9	-0.3
Proline	177.0	9.0	170.4	14.7	-3.8
Threonine	119.5	6.1	104.5	10.4	-12.6
Histidine	72.6	3.6	72.7	6.7	0.1
Arginine	81.4	2.3	91.7	8.4	12.6
Lysine	140.0	14.0	140.5	8.9	0.1

Precision: Inter-assay precision was assessed by measuring 6 replicates of the reference plasma (NIST 1950) once a week for 4 weeks (n = 24) Intra-assay coefficients of variation (CVs) for BCAAs were in the range of 3.0 – 6.5 % for fixed reference concentrations while inter-assay CVs ranged from 6.3 to 10.4 %.

Intra- and Inter-assay precision assessment for AA measurements			
Analyte	Concentration [mg/L] (SD)	Intra-assay CV n = 6 [%]	Inter-assay CV n = 24 [%]
Leucine	13.17 (0.84)	6.1	10.3
Phenylalanine	8.37 (1.12)	3.3	6.5
Isoleucine	7.28 (0.43)	5.8	10.4
Methionine	3.33 (0.27)	3.9	8.1
Valine	21.34 (1.21)	5.7	8.8
Proline	20.31 (1.12)	2.5	8.6
Threonine	14.23 (0.71)	6.5	9.9
Histidine	11.27 (0.56)	4.5	9.2
Arginine	14.18 (0.41)	3.5	9.1
Lysine	20.42 (1.94)	3.0	6.3
Tryptophan	9.64 (0.76)	4.4	7.9
1-Methylhistidine	0.88 (0.08)	2.0	8.8
3-Methylhistidine	1.34 (0.13)	2.6	9.9

Supplemental Table S3: Correlations between fat mass and biomarkers

	Fat mass (kg)	P-value
**Visceral adipose tissue** [1]		
MDA	0.662	**0.010**
**Subcutaneous adipose tissue** [1]		
MDA	0.647	**0.012**
Retinol [µmol/kg]	0.555	**0.039**
**Plasma** [2]		
β-Carotene	-0.384	**0.040**
Retinol	-0.558	**0.002**
Retinol/Cholesterol	-0.369	**0.049**
Cholesterol	-0.412	**0.026**
Lysine [µM]	0.389	**0.037**
1-MH	-0.468	**0.011**

[1] n = 14, [2] n = 29; Pearson correlation (transformed values were used)

Supplemental Figure S1: Correlation between MDA tissue concentrations and fat mass

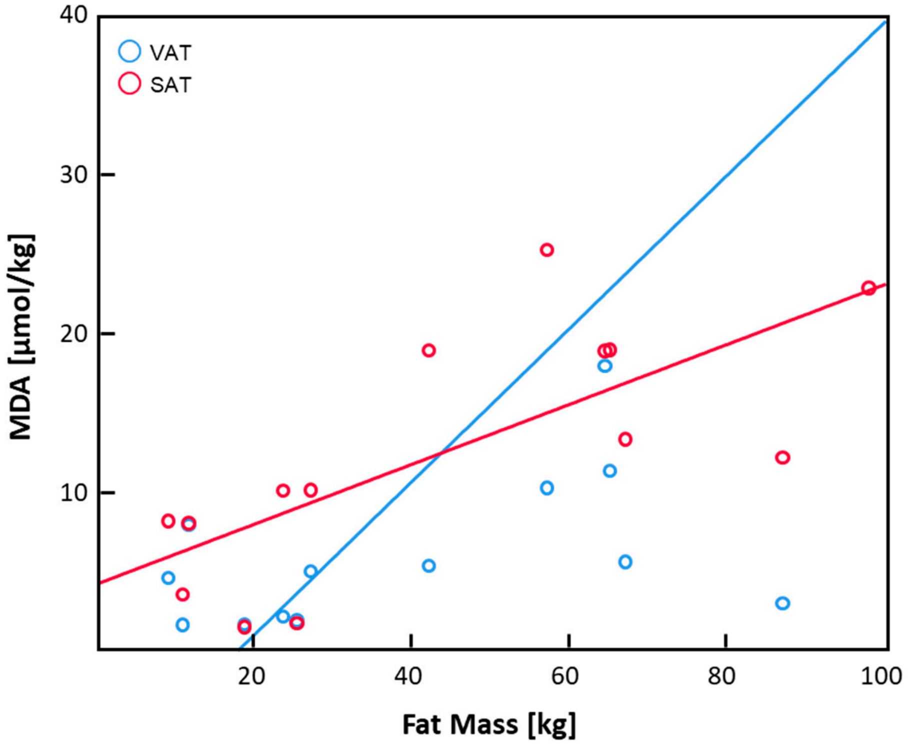

Supplement: Supplementary file 1 [file antioxidants-11-00774-s001.zip › antioxidants-1658657-supplementary.pdf]
